# Fulminant Klebssiella Pneumoniae Pneumonia in Immunocompetent Non Alcoholic Patient

**DOI:** 10.4137/ccrep.s761

**Published:** 2008-06-10

**Authors:** Z. Benyashvili, A. Djirbe, N. Assy

**Affiliations:** 1 Department of Internal Medicine, Sieff Government Hospital, Safed, Israel.; 2 Liver Unit, Sieff Government Hospital, Safed, Israel.; 3 Technion Institute, Faculty of Medicine, Haifa, Israel.

**Keywords:** pneumonia, klebssiella, bacteremia, hyperdense infiltrates, bulging interlobar fissure, death

We describe a case of fulminant community acquired bacteremic K. pneumoniae pneumonia in non alcoholic (<20 gr/day) patient. A previously healthy 36-year-old man presented with fever (39 C), malaise, dyspnea and a nonproductive cough. On physical examination, he had tachycardia (120/min), tachypnea (31/min) and hypotension (85/67 mm Hg). A chest radiograph displayed symmetric bilateral and voluminous hyperdense lung consolidation with bulging interlobar fissure (Fig. 1A). Blood work showed neutropenia (WBC 3000 counts/ml, Neutrophiles 400), metabolic acidosis (PH 7.1, bicarbonate 10, PO_2_ 70, PCO_2_ 23) and acute renal failure (Cr 3.8, BUN 76). Within a few hours, the patient’s condition deteriorated with sudden onset of acute respiratory distress and shock with disseminated intravascular coagulation (platelets 70,000 counts/ml, D-Dimmers 15, fibrinogen 180 mg/dL, INR 1.5). Despite ventilatory support and the administration of intravenous fluids, Granulocyte-Macrophage Colony Stimulating Factor, antibiotics (cefuroxime and roxithromycin), and vasopressive agents, the patient died within a few hours.

One of two blood cultures later revealed Kliebssiella pneumoniae. Autopsy showed voluminous inflammatory lung exudate with massive neutrophile infiltrates fibrin and lung edema (Fig. 1B). Klebsiella pneumoniae pneumonia is an uncommon community-acquired pneumonia but common nosocomial infection. Only four cases of community-acquired bacteremic Klebsiella pneumoniae pneumonia were reported in the 2-year study period in the united state, Argentina, Europe, or Australia; and none were in alcoholics. In contrast, 53 cases of bacteremic Klebsiella pneumoniae pneumonia were observed in South Africa and Taiwan, where an association with alcoholism was observed (1). Three prominent presentations of community–acquired klebsiella infection has been described. First, toxic presentation with sudden onset, high fever, and hemoptysis, chest radiographic abnormalities such as bulging inter-lobar fissure and cavitary abscesses are prominent (2). Second, invasive presentation of K. pneumoniae infection with liver abscess has been described in Asia (3). The third striking clinical observation is the preponderance of K. pneumoniae as a cause of community acquired bacterial meningitis in adults in Taiwan, even in the absence of liver abscess or other sites of infection (4). Our case belongs to the first advanced clinical presentation with the formation of voluminous inflammatory exudates leading to lobar expansion with resulting bulging of interlobar fissures.

Severe gram-negative infection and gram negative bacteremia are the leading causes of sepsis and septic shock (5). In this disease process, the pathogen and the host’s immune response may trigger a cascade of pathophysiologic responses that results in mutiorgan system failure. Lipopolysaccharide, a component of the outer membrane of gram negative bacteria, is able to stimulate the production and release of cytokines including TNF-alpha, Interleukin-1, IL-6 and IL-8. These factors have been shown to produce a syndrome similar to sepsis with fever, tachycardia, and hypotension (6). Several cytotoxic products, including oxygen free radicals and lysosomal enzymes, are manufactured, resulting in pathogen and host cell damage. Concomitantly, hypotension and shock are mediated by a loss of vascular tone in arteriolar smooth muscle and capillary endothelial hyper permeability leading to leakage of intravascular volume into the extra vascular space (6). A diagnosis of ARDS was excluded by clinical and pathological findings. Ratio of PO_2_ to inspired fraction of oxygen was more than 200, and there was intense neutrophilic infiltration in the interstitium and intra-alveolar spaces but no diffuse alveolar damage with hyaline membranes or endothelial cell necrosis, edema, or organizing interstitial fibrosis (7).

Despites advances in antimicrobial therapy and medical technology, sepsis continues to lead to more than 100,000 deaths per year with a mortality of 40% (8). Bad prognostic factors of community acquired pneumonia in patients admitted to the hospital include septic shock, Klebsiella pneumoniae, and bacteremia (9). In conclusion, diagnosis of bacteremic klebsiella pneumoniae pneumonia may be difficult in the absence of underlying disease. Therefore, Klebsiella pneumonia should be considered when the chest x-ray demonstrates a voluminous hyper dense lung consolidation with multi systemic organ failure. Early diagnosis and appropriate treatment of klebsiella pneumoniae pneumonia (ceftriaxone and azithromycin) are mandatory in order to improve prognosis. The molecular epidemiology data and information regarding the genomic background of the strains such as the presence or absence of the kfu/PTS region is ongoing.

## Figures and Tables

**Figure 1:**
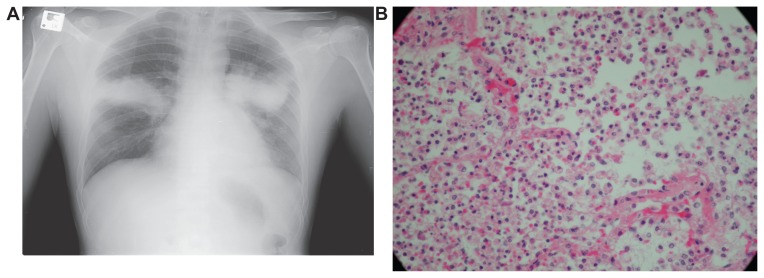
Radiological (**A**) and pathological (**B**) representation of Klebssiella Pneumonia: Note the prominent voluminous hyperdense, symmetric bilateral lung consolidation and the massive infiltration of neutrophils in the interstitium and intra alveolar spaces.
